# Isolation and characterization of salt-tolerant bacteria with plant growth-promoting activities from saline agricultural fields of Haryana, India

**DOI:** 10.1186/s43141-021-00186-3

**Published:** 2021-06-28

**Authors:** Arti Sharma, Kamal Dev, Anuradha Sourirajan, Madhu Choudhary

**Affiliations:** 1 Faculty of Applied Sciences and Biotechnology, Shoolini University, Bajhol, PO 173229, District Solan, Sultanpur, Himachal Pradesh India; 2 ICAR-Central Soil Salinity Research Institute (CSSRI), Karnal, 132001 India

**Keywords:** Ammonia, HCN, Indole acetic acid, Phosphate solubilization, PGPR, Salt tolerant

## Abstract

**Background:**

Soil salinity has been one of the biggest hurdles in achieving better crop yield and quality. Plant growth-promoting rhizobacteria (PGPR) are the symbiotic heterogeneous bacteria that play an important role in the recycling of plant nutrients through phytostimulation and phytoremediation. In this study, bacterial isolates were isolated from salt-polluted soil of Jhajjar and Panipat districts of Haryana, India. The potential salt-tolerant bacteria were screened for their PGPR activities such as phosphate solubilization, hydrogen cyanide (HCN), indole acetic acid (IAA) and ammonia production. The molecular characterization of potent isolates with salt tolerance and PGPR activity was done by 16S rDNA sequencing.

**Results:**

Eighteen soil samples from saline soils of Haryana state were screened for salt-tolerant bacteria. The bacterial isolates were analyzed for salt tolerance ranging from 2 to 10%. Thirteen isolates were found salt tolerant at varied salt concentrations. Isolates HB6P2 and HB6J2 showed maximum tolerance to salts at 10% followed by HB4A1, HB4N3 and HB8P1. All the salt-tolerant bacterial isolates showed HCN production with maximum production by HB6J2. Phosphate solubilization was demonstrated by three isolates viz., HB4N3, HB6P2 and HB6J2. IAA production was maximum in HB4A1 (15.89) and HB6P2 (14.01) and least in HB4N3 (8.91). Ammonia production was maximum in HB6P2 (12.3) and least in HB8P1 (6.2). Three isolates HB6J2, HB8P1 and HB4N3 with significant salt tolerance, and PGPR ability were identified through sequencing of amplified 16SrRNA gene and were found to be *Bacillus paramycoides*, *Bacillus amyloliquefaciens* and *Bacillus pumilus*, respectively.

**Conclusions:**

The salt-tolerant plant growth-promoting rhizobacteria (PGPR) isolated from saline soil can be used to overcome the detrimental effects of salt stress on plants, with beneficial effects of physiological functions of plants such as growth and yield, and overcome disease resistance. Therefore, application of microbial inoculants to alleviate stresses and enhance yield in plants could be a low cost and environmental friendly option for the management of saline soil for better crop productivity.

**Supplementary Information:**

The online version contains supplementary material available at 10.1186/s43141-021-00186-3.

## Background

The world population is estimated approximately 7.8 billion. This is projected to rise up to 9.7 billion by 2050. The increase in the world’s population has increased the demand of food products [1, 2]. However, crop production per unit of land cultivated is unequal to meet the desired demand of food. The change in climate, loss of soil structure, nutrient degradation, draught and soil salinity are the major factors behind the decreased crop yield [2–4]. A worldwide loss of 50% land of the total land mass has been estimated by Food and Agricultural Organization (FAO) by the year 2050. Excess salt concentration in soil has negative impacts on plant growth and metabolism [5]. The salinity stress may also lead to generation of free radicals such as superoxide ions, hydrogen peroxide (H_2_O_2_), and singlet oxygen, decrease in plant defensive enzymes, imbalance in sodium hemostasis, decreased iron uptake, phenols and other trace elements [6–8].

In the recent time, various approaches have been applied to solve the problem of soil salinity and acidity [9–12]. There are some alternative methods available for retrieval of salt-affected soils such as phytoremediation and bioremediation [13–15]. The plant growth-promoting rhizobacteria (PGPR) are the heterogenous bacteria which are well known for their beneficial activities. There are a number of rhizobacteria such as genera of *Alcaligenes*, *Pseudomonas*, *Azospirillum*, *Bacillus*, *Klebsiella*, *Azotobacter*, *Enterobacter*, *Burkholderia*, *Arthrobacter* and *Serratia* that aid in plant growth through various mechanisms [16–18]. These bacteria act as biofertilizers and play an important role in the recycling of plant nutrients which help in phytostimulation and phytoremediation [19]. In the present study, bacteria were isolated from saline soils and screened for salt tolerance and PGP activities. PGP bacteria not only increase production of exopolysaccharides, siderophores, alter pH, modify toxic metals and solubilize phosphorus but also help in evacuating stress-alleviating metabolite 1-aminocyclopropane-1-carboxylic acid deaminase. They also play an important role in the secretion of indole-3-acetic acid (IAA), cytokinin and gibberellins and the development of antibiotic resistance [20–26].

## Methods

### Collection of soil samples

**Fig. 1:**
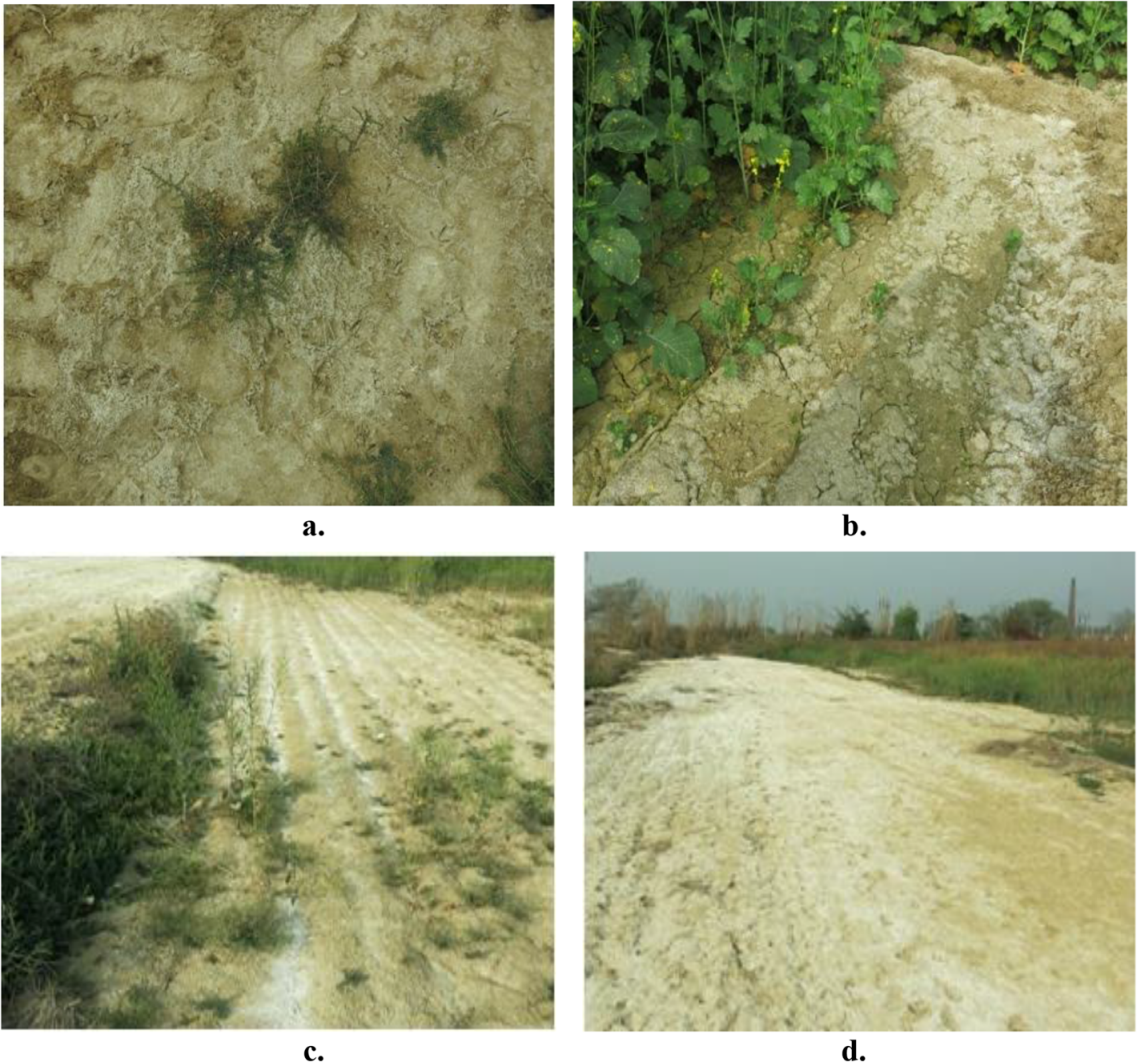
Saline soil fields of Jhajjar, Haryana, India (**a** and **b**) and Panipat, Haryana (**c** and **d**)

A total of 18 soil samples were collected from saline soils of Jhajjar and Panipat districts of Haryana. Ten out of eighteen soil samples were collected from the waterlogged saline area of the Jhajjar district of Haryana (28.37° North, 76.39° East). The remaining eight soil samples were collected from ICAR-CSSRI Nain farm, Panipat, Haryana (29.39° North, 76.97° East) (Fig. 1). All samples were collected in sterile polybags and divided in two parts, one for chemical analysis and second for isolation of PGP bacteria. Second part of the soil was stored at 4 °C till isolation.

### Analysis of soil samples for pH and electrical conductivity

Soil samples were sieved by a 2-mm sieve and dried before analysis. Soil pH and electrical conductivity (EC) in soil:water ratios of 1:2 was determined as per the standard method described by Jackson [27].

### Isolation and screening of bacteria for salt tolerance from saline soils

The isolation of salt-tolerant bacteria was done from the soil samples on different types of selective media such as Ashbys Mannitol Agar for Azotobacter and nonselective media such as Tryptone soya agar, Pikovasky’s Agar for phosphate-solubilizing bacteria and Jenson media for nitrogen-fixing bacteria [28]. The serial dilutions of the samples were plated on selected media and incubated for 48–72 h at 25 ± 2 °C. The pure culture of bacteria was obtained by repeated sub-culturing. The isolates were tested for their salt tolerance ability by spot inoculating the isolates on nutrient broth containing different concentrations of NaCl (2%, 4%, 6%, 8% and 10%), incubated at 25 ± 2 °C for 5 days and observed for growth.

### Characterization of salt-tolerant bacterial isolates for plant growth-promoting activity

The isolates were further screened for PGP activities such as phosphate solubilization, production of indole acetic acid, hydrogen cyanide (HCN) and ammonia.

#### Phosphate solubilization

Phosphorus solubilizing activity of isolates was determined qualitatively according to the method described by Nautiyal [29]. Pikovskaya’s agar medium containing calcium tri phosphate (0.5%) as the inorganic form of phosphate was used in assay, 2.5 μl of the bacterial culture (O.D. 600) was streaked on the plates. All the plates were incubated at 28 °C for 4–5 days. Transparent halo zone around the bacterial colony indicates the phosphate solubilizing activity of the bacterial isolates.

#### Production of indole acetic acid (IAA)

Bacterial isolates were inoculated in nutrient broth enriched with tryptophan (1–2%) and incubated for 24 h at 28 °C on rotary shaker. Cultures were centrifuged at 10,000 rpm for 15 min. Salkowski’s reagent was mixed with supernatant, and incubated for 25 min at room temperature. Development of the pink color indicated IAA production and the quantitative estimation of IAA were performed by using the method described by Gordon and Weber [30].

#### Production of HCN

The salt-tolerant bacterial isolates were streaked on nutrient Agar medium containing 4.4 g/l glycine. Filter paper discs were dipped in 0.5% picric acid prepared in 2% sodium carbonate. The discs were placed in the lid of each petri plate and sealed. All the plates were incubated for 4 days at 28 °C. Color change of the filter paper from deep yellow to orange and orange to brown indicated the production of HCN by the bacterial isolate [31].

#### Production of ammonia

The salt-tolerant bacterial isolates were cultured in peptone water at 30 °C for 4 days. One milliliter of Nessler’s reagent was added to each tube. Development of faint yellow color indicated small amounts of ammonia production, and deep yellow to brownish color indicated maximum ammonia production [32].

#### Molecular characterization of bacterial isolates

##### Isolation of genomic DNA

Bacterial genomic DNA was isolated by taking 24-h-old broth culture (10 ml) of bacterial isolates. The broth culture was centrifuged at 2000 rpm for 10 min, and supernatant was discarded. One milliliter of freshly prepared extraction buffer was added to the pellet, and the resulting solution was transferred in a 2-ml Eppendorf tube, incubated at 65 °C in a water bath for 30 min, and equal volume of ice-cold solution of phenol: chloroform: isoamyl alcohol (25:24:1) was added to it. The solution was mixed well and centrifuged at 10,000 rpm for 10 min at 4 °C, and the upper aqueous layer was transferred to a new Eppendorf tube. Equal volume of ice-cold solution of phenol: chloroform: isoamyl alcohol (25:24:1) was added to this aqueous layer, and the step was repeated 3–4 times. In the final aqueous layer, the ice-cold absolute alcohol was added in excess to precipitate the bacterial gemomic DNA and the solution was centrifuged at 10,000 rpm at 4 °C for 10 min. The supernatant was discarded, and the pellet was washed with 100 μl of 70% (v/v) ethanol and centrifuged at 10,000 rpm for 15 min at 4 °C, the supernatant was discarded, and the pellet was dissolved in 50 μl of TE buffer (pH 8.0) and stored at – 20 °C for further studies.

##### Amplification and sequencing of 16S rRNA

Universal primers were used for the amplification of the 16S rRNA sequence of the selected salt-tolerant PGP isolates. The amplified product was purified and sequenced from Eurofins Genomics India Pvt. Ltd, Bengaluru. The sequence obtained were analysed and identified using BLAST search and were compared against bacterial 16S rRNA sequence available on NCBI database. The sequences were aligned by using Clustal W 1.74 followed by construction of neighbour joining phylogenetic tree, using MEGA4.

## Results

### Isolation of bacteria from saline soils of Haryana, India

In the present study, a total of eighteen soil samples from saline soils of Jhajjar and Panipat districts of Haryana state were screened for salt-tolerant bacteria (Fig. 1). Soil samples were evaluated for their pH and electrical conductivity. In Jhajjar district’s soil samples, electrical conductivity was maximum in HB5 (16.56) and minimum in HB9 (5.40). The pH of different soil samples varied from 8.61 (HB8) to 6.50 (HB1). In Panipat district’s soil samples, electrical conductivity was maximum in PS5 (10.01) and minimum in PS4 (2.33), whereas pH value ranged from 9.0 (PS5) to 8.21 (PS4) (Table 1). A total of 81 bacterial isolates were isolated from ten soil samples collected from salt-affected land of Jhajjar (Table 2). Sixteen isolates each were isolated from soil samples HB4 and HB6, whereas none of the bacteria were isolated from HB1. Maximum number of isolates was isolated on nutrient agar plates and least on Kings B agar media (Table 2). Twenty-six bacterial isolates from eight soil samples were isolated from soil samples of Panipat district (Table 3). Four isolates were isolated from each of PS2, PS4 and PS5, three isolated from PS5, PS6, PS7 and PS8 and two isolated from PS1. Maximum numbers of isolates were isolated on nutrient agar plates and least numbers on Ashby’s agar (Table 3).

**Table 1:** Soil samples collected from Jhajjar (serial nos. 1 to 10) and Panipat districts (serial nos. 11–18) of Haryana, India along with their EC and pH analysis

Sr. no.	Soil sample	Electrical conductivity (EC) dS/m	pH
1	**HB1**	13.84	6.50
2	**HB2**	14.26	6.65
3	**HB3**	13.96	6.72
4	**HB4**	15.32	6.90
5	**HB5**	16.56	6.84
6	**HB6**	11.30	7.5
7	**HB7**	10.92	8.12
8	**HB8**	12.70	8.61
9	**HB9**	5.40	7.9
10	**HB10**	10.62	8.27
11	**PS1**	5.04	8.90
12	**PS2**	4.74	8.96
13	**PS3**	3.04	8.24
14	**PS4**	2.33	8.21
15	**PS5**	10.01	9.00
16	**PS6**	7.90	8.80
17	**PS7**	12.10	8.86
18	**PS8**	11.53	8.97

**Table 2:** Details of bacteria isolated from soil samples of Jhajjar, Haryana, India

S. no.	Soil sample	Growth of bacterial isolates on different media used						No. of bacterial isolates
		Nutrient agar (N)	Jensen’s agar (J)	Pikovaskaya agar (P)	King’s B agar (K)	Ashby’s agar (A)	Triple sugar agar (T)	
1	**HB1**	Undetected	Undetected	Undetected	Undetected	Undetected	Undetected	0
2	**HB2**	Undetected	Undetected	HB2P2	Undetected	Undetected	HB2T1	2
3	**HB3**	HB3N1, HB3N2 HB3N3, HB3N4	HB3J1, HB3J2	HB3P1 HB3P2	Undetected	HB3A1	HB3T1	10
4	**HB4**	HB4N1, HB4N2 HB4N3, HB4N4 HB4N5	HB4J1, HB4J2 HB4J3, HB4J4	Undetected	Undetected	HB4A1	HB4T1, HB4T2 HB4T3, HB4T4 HB4T5, HB4T6	16
5	**HB5**	HB5N1, HB5N2	Undetected	Undetected	Undetected	Undetected	HB5T1, HB5T2	4
6	**HB6**	HB6N1, HB6N2 HB6N3	HB6J1, HB6J2	HB6P1, HB6P2 HB6P3	Undetected	HB6A1, HB6A2	HB6T1, HB6T2 HB6T3, HB6T4 HB6T5, HB6T6	16
7	**HB7**	HB7N1, HB7N2 HB7N3	Undetected	HB7P1	Undetected	HB7A1	HB7T1, HB7T2 HB7T3	8
8	**HB8**	HB8N1, HB8N2 HB8N3	HB8J1, HB8J2	HB8P1	Undetected	HB8A1, HB8A2	HB8T1, HB8T2	10
9	**HB9**	HB9N1 HB9N2	HB9J1 HB9J2	HB9P1 HB9P2	Undetected	HB9A1, HB9A2 HB9A3, HB9A4	HB9T1	11
10	**HB10**	HB10N1, HB10N2	HBI0J1, HBI0J2	Undetected	Undetected	Undetected	Undetected	4
**Total no. of isolates**		24	14	10	0	11	22	**81**

**Table 3:** Details of bacteria isolated from soil samples of Panipat, Haryana, India

Sr. no.	Soil samples	Growth of bacterial isolates on different media used						No. of bacterial isolates
		Nutrient agar (N)	Jensen’s agar (J)	Pikovaskaya agar (P)	King’s B agar (K)	Ashby’s agar (A)	Triple sugar agar (T)	
1	**PS1**	PS1N1	Undetected	Undetected	PS1K1	Undetected	Undetected	2
2	**PS2**	PS2N2	PS2J2	Undetected	PS2K1 PS2K2	Undetected	Undetected	4
3	**PS3**	PS3N1	PS3P1	Undetected	PS3K1	Undetected	Undetected	3
4	**PS4**	PS4N1	Undetected	PS4P1	PS4K1	Undetected	PS4T1	4
5	**PS5**	PS5N1	Undetected	PS5P1	PS5K1	PS5A1	Undetected	4
6	**PS6**	PS6N1	PS6J1	Undetected	PS6K1	Undetected	Undetected	3
7	**PS7**	PS7N1	Undetected	Undetected	PS7K1	Undetected	PS7T1	3
8	**PS8**	PS8N1	PS8J1	PS8P1	Undetected	Undetected	Undetected	3
**Total no. of isolates**		8	4	3	8	1	2	**26**

### Screening of bacteria isolates for salt tolerance and PGPR potential

**Fig. 2:**
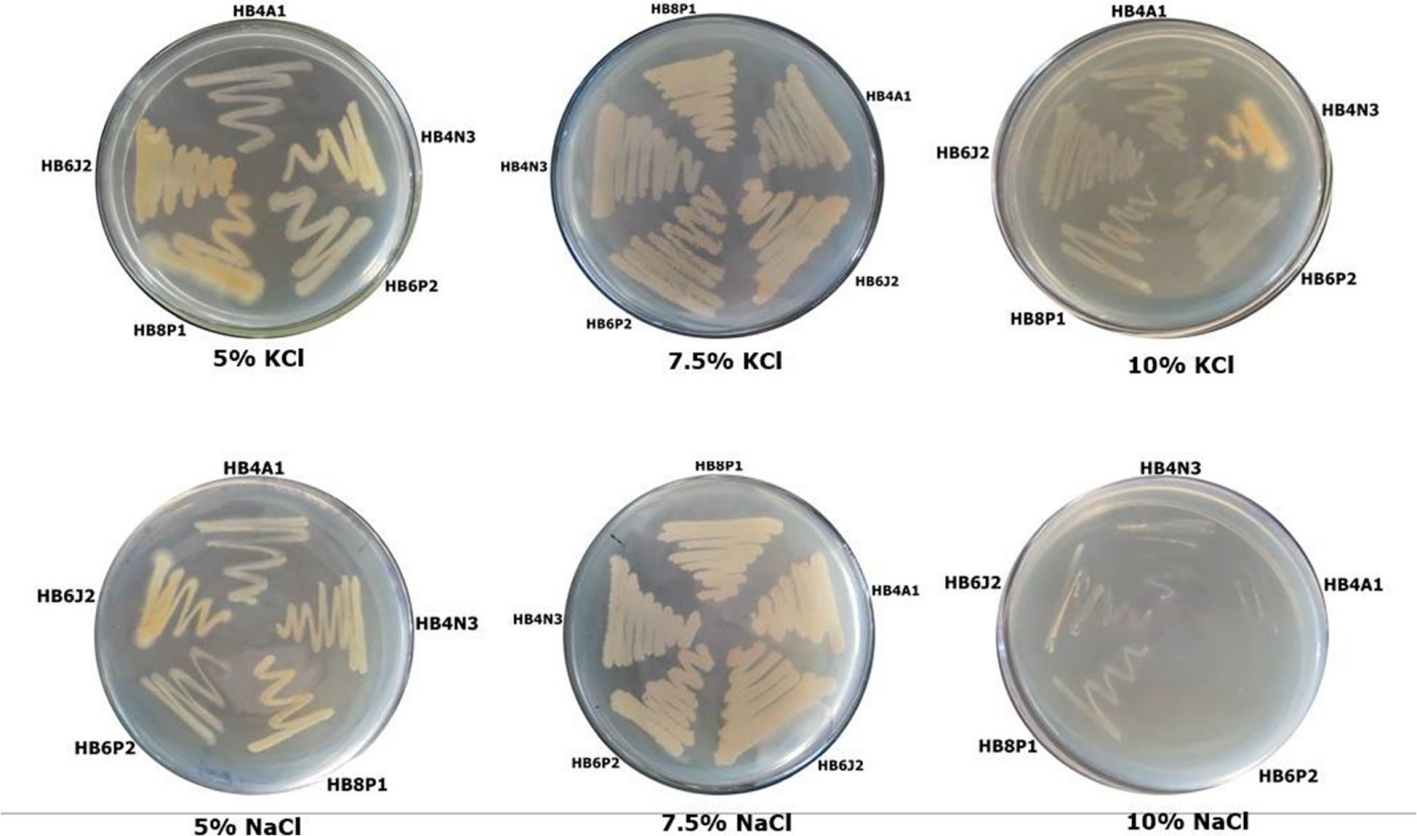
Growth of bacterial isolates HB6J2, HB8P1, HB4A1, HB4N3 and Hb6P2 on media containing 5%, 7.5% and 10% KCl, and NaCl, respectively

A total of thirteen isolates were found salt tolerant at varied salt concentrations. Isolates HB6P2 and HB6J2 have shown maximum tolerance to salts at 10% concentration followed by HB4A1, HB4N3 and HB8P1. All the isolates have shown salt tolerance up to 7.5% salt concentration; however, only five isolated bacteria were able to grow at 10% concentration (Fig. 2). The salt-tolerant bacteria were further screened for the ability to produce various plant growth-promoting traits such as HCN production, phosphate solubilization, IAA and ammonia production (Additional file 1: Fig. S1 and S2; Table 4). All the salt-tolerant bacteria demonstrated PGPR activities (Table 4). The phosphate solubilization was demonstrated by three salt-tolerant bacterial isolates viz; HB4N3, HB6P2 and HB6J2. The IAA production was maximum in HB4A1 (15.89) and HB6J2 (15.84) and least in HB4N3 (8.91). Ammonia production was maximum in HB6P2 (12.3) and least in HB8P1 (6.2) (Table 4).

**Table 4:** Plant growth promoting (PGPR) activities of salt tolerant bacterial isolates

Sr. no.	Isolate	Growth on media with 10% KCl & NaCl	HCN production activity	Phosphate solubilization activity	Production of IAA/ ammonia (in ppm)	
					IAA	Ammonia
1	**HB4A1**	+	+	Undetected	15.89	9.1
2	**HB4N3**	+ +	+	+	8.91	10.5
3	**HB6P2**	++	+	+	14.01	12.3
4	**HB6J2**	++	+++	+	15.89	9.1
5	**HB8P1**	+	+	Undetected	9.57	6.2

+: low; ++: medium; +++: high

### Molecular identification of salt-tolerant bacterial isolates with PGPR activities

**Fig. 3:**
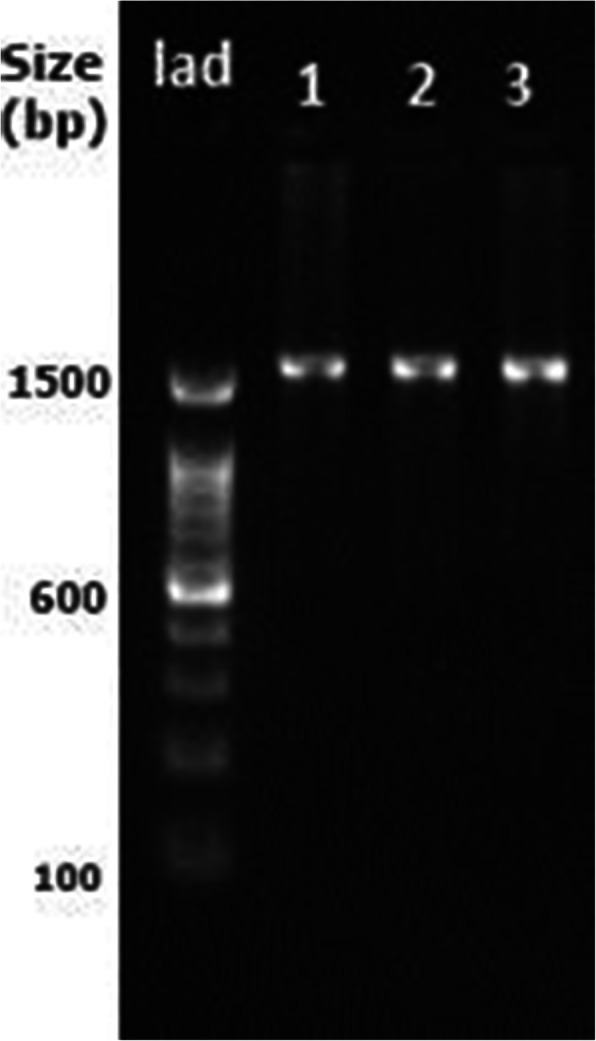
PCR amplified product of 16S rDNA of bacterial isolates. (Lad: molecular size marker 100 bp; lane 1: isolate HB6J2; lane 2: HB8P1; lane 3: HB4N3)

**Fig. 4:**
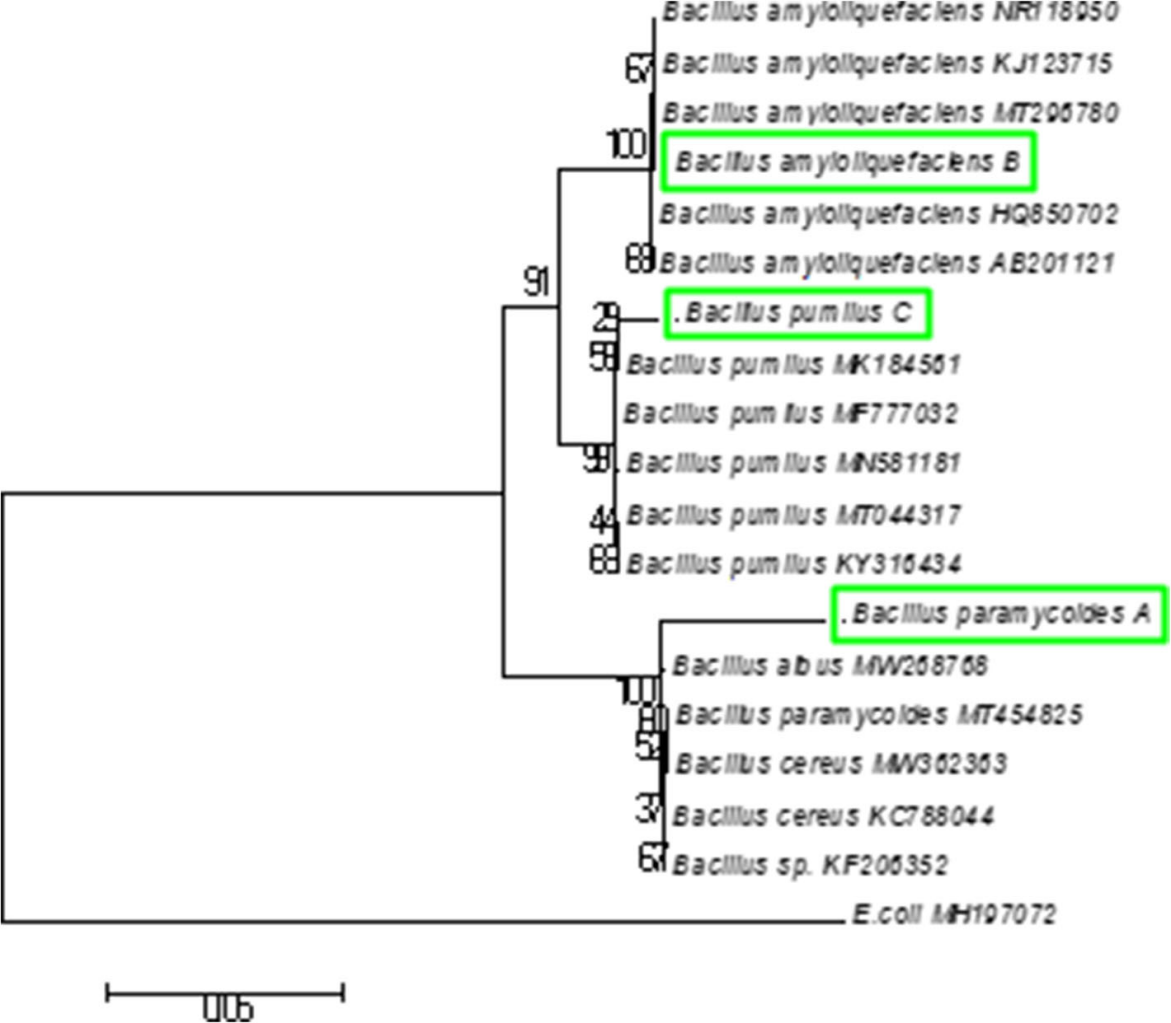
Phylogenetic tree constructed by the neighbor-joining method derived from analysis of the 16S rRNA gene sequence of salt-tolerant PGPR bacterial isolates and related sequences obtained from NCBI. Scale bar, 0.05 substitutions per nucleotide position. *Bacillus paramycoides* **A** (HB6J2), *Bacillus amylofaciens* **B** (HB8P1) and *Bacillus pumilus* **C** (HB4N3)

Identification of the salt-tolerant PGPR isolates was based on PCR amplification of 16S rRNA gene sequences The PGPR isolates HB6J2, HB8P1 and HB4N3 were further selected for molecular characterization based on their promising salt tolerance and plant growth-promoting properties. The 16S rRNA gene of the selected isolates was successfully amplified using PCR, and approximately 1500 bp of the amplified products were sequenced (Fig. 3). The BLAST-N comparison of the searched sequences in the NCBI nucleotide database revealed 99.87% similarity of the isolate HB6J2 with *Bacillus paramycoides* M9a1a (NCBI accession number: MT454825.1), 99.30% similarity of HB8P1 with *Bacillus amyloliquefaciens* strain K-8 (NCBI accession number: MT296780.1) and 98.88% similarity of HB4N3 with *Bacillus pumilus* strain EE107-PS (NCBI accession number: MN581181.1) (Additional file 1: Fig S3-S5). The phylogenetic tree of the selected bacterial isolates was constructed by using the neighbor-joining method (Fig. 4).

## Discussion

The increase in soil salinization is one of the most common environmental threats for crop yield and quality [33, 34]. This problem has been regarded as one of the major hurdles in crop productivity in arid and semiarid regions [35, 36]. Sodic and saline soil is reducing the cultivable area for agriculture by 1–2% every year, thereby reducing food production [37, 38]. In the present study, all the soil samples collected from Panipat district are alkaline compared with soil samples of Jhajjar, while most of the Jhajjar soil samples have higher electrical conductivity than soil samples of Panipat (Table 1). The alkaline nature of the soil may affect the nutritional availability for plant growth. These variations in pH and EC might be responsible for the poor soil structure and infertility of the sample collection sites [2].

In the present study, a total of thirteen isolates were found salt tolerant at different salt concentrations. Isolates HB6P2 and HB6J2 have shown maximum tolerance to salts at 10% concentration followed by HB4A1, HB4N3 and HB8P1. In a similar study conducted by Mahmood and co-workers (2019), isolated, screened and characterized rhizosphere bacteria from the common ice plant *Mesembryanthemum crystallinum* L. In their study, 80 isolated strains out of 152 demonstrated tolerance to soil salinity. Bacterial strains of *Streptomyces* sp. PR-3 and *Bacillus* sp. PR-6 were effective against soil salinity (< 1250 mM NaCl) [39]**.** Zerrouk et al. [40], evaluated the capacity of *Pseudomonas plecoglossicida* strain Pp20 to mitigate the damages on maize roots caused by salt and aluminum. These workers found that Pp20 had the ability to grow at varied NaCl concentrations ranging from 50 to 600 mM. Their findings revealed a positive impact on stem weight, seminal roots, lateral roots and root length [41]. Similarly, study conducted by Ma and co-workers (2019), demonstrated the impact of *Pseudomonas libanensis* TR1 on *Helianthus annuus* and *P. libanensis* in exhibiting high resistance against saline stress (8%) [40]. However, in our study, all the isolated bacteria have shown salt tolerance up to 7.5% salt concentration, and only five isolates were able to grow at 10% concentration (Fig. 2). Several other workers tested *Bacillus* SB1 and *Halobacillus* SB2 strains for salt tolerance studies in combination with metals such zinc, aluminium and lead in the growth of *Arachis hypogaea* L under saline stress [42]. However, in our study, we did not perform salt tolerance studies in combination with metals.

The primary concern regarding saline soil is its impact on plant growth. Excessive salt concentration (more than 200 mM) may inhibit plant growth [43, 44]. In the present study, the bacterial isolates were further tested for their tolerance to salts at different salt concentrations ranging from 5 to 10% concentration (Fig. 2). The salt-tolerant bacteria were further screened for the ability to produce various plant growth-promoting traits such as HCN production, phosphate solubilization, IAA and ammonia production, and in the present study, all the salt-tolerant bacteria demonstrated PGPR activities (Table 4). In a similar study, conducted by Noori et al. [45], also isolated rhizobial and non-rhizobial drought and salinity-tolerant bacteria from the surface sterilized root nodules of alfalfa, grown in saline soils, and evaluated the effects of potential isolates on plant growth under salt stress. These workers co-inoculated alfalfa plant with bacterial strains such as *Klebsiella* sp. A36, *K. cowanii* A37, and rhizobial strain *S. meliloti* ARh29. In their results, it was demonstrated that *Klebsiella* sp. A36, *Kcowanii* A37 could deliver plant nitrogen and upsurge plant growth indices without rhizobial bacteria and nitrogen [46]. In the present study, phosphate solubilization was demonstrated by three salt-tolerant bacterial isolates viz; HB4N3, HB6P2 and HB6J2 (Table 4). In a similar study, *Bacillus* sp. (SB1) and *Halobacillus* sp. (SB2) isolated from groundnut rhizosphere had shown the ability to overcome the salt and metal stress [42]. In another study, the soil salinity mitigation by *Streptomyces* sp. strain PR-3 was done through phosphorus solubilization. These workers also demonstrated siderophore production by *Bacillus* sp. strain PR-6 and PR-3 and IAA production by PR-6 strain and suggested that these bacteria have the ability to promote growth in the common ice-plant [45].

Komaresofla et al. [47], demonstrated improved growth and salinity tolerance of the halophyte *Salicornia sp.* by co-inoculation with endophytic and rhizosphere bacteria. These workers evaluated drought tolerance of salt-tolerant isolates by performing 1-aminocyclopropane-1-carboxylate (ACC)-deaminase, IAA production and phosphate solubilization assays [47]. In the present study, all the isolated salt-tolerant bacteria have shown HCN production with maximum production by HB632 isolate. It was revealed that plant growth-promoting microbial isolates demonstrated escalation in ascorbate peroxidase (APX), superoxide dismutase (SOD), catalase (CAT) and glutathione peroxidase (GPX) and antioxidative enzymes under saline environment [48]. Sarkar et al. [48], studied halotolerant *Enterobacter* sp. strain P53 inoculated with rice seedlings. In their study, the bacteria demonstrated production of IAA, HCN, siderophore and antioxidant activity under salt stress [39]. We also obtained similar findings in our study; all the salt-tolerant bacterial isolates showed PGPR activities (Table 4).

The isolates HB6J2, HB8P1 and HB4N3 with significant salt-tolerant and PGPR ability were sequenced for amplified 16SrRNA gene and identified as *Bacillus paramycoides*, *Bacillus amyloliquefaciens* and *Bacillus pumilus* respectively. In a similar study conducted by Sultana et al. [49], three salt-tolerant bacterial isolates viz; *Bacillus aryabhattai*, *Achromobacter denitrificans*, and *Ochrobactrum intermedium* were identified through comparison of 16S rRNA gene sequences. These bacteria exhibited high atmospheric nitrogen fixation, phosphate solubilization, and indoleacetic acid production at concentration of 200 mmol/l salt [49]. In another study conducted by Tripathi et al. [50] studied the diversity of salt-tolerant bacteria present in the rhizosphere of *Oryza sativa*. These workers isolated fourteen bacterial isolates showing tolerance to 3% NaCl; however, these workers used restriction patterns produced by amplified 16S rDNA after digestion with restriction enzymes such as *Sau*3AI, *Alu*I and *Rsa*I for molecular characterization of bacterial isolates. Also, biodiversity among the strains was analyzed through the application of random amplified polymorphic DNA RAPD technique [50]. The salt-tolerant bacteria with PGPR activities may prove beneficial in the management of salt-affected agricultural fields for crop improvement. Alternatively, halophilic bacteria and their genes can be mined for salt-tolerant PGPR activities or salt tolerance traits can be transferred to crop plants [51–53].

## Conclusion

The continuous increase in the population has also created more demand of food and hence agriculture productivity. Soil salinity has been one of the largest barriers in achieving better crop yield and quality. The much cost-effective methods such as application of microbes having PGPR activities can enhance plant growth, speed up seed germination, improve seedling emergence and protect plants. In the present study, a total of 18 soil samples from saline soils of Haryana state were screened for salt-tolerant bacteria. The bacterial isolates were further tested for their tolerance to different salt concentrations, and a total of thirteen isolates were found salt tolerant at varied salt concentrations. All the isolated salt-tolerant bacteria were further tested for the ability to produce PGPR activities such as phosphate solubilization, HCN, IAA and ammonia production. All the salt-tolerant bacteria demonstrated significant PGPR activities. The three isolates viz; HB6J2, HB8P1 and HB4N3 with good PGPR activities were identified, though sequencing of amplified 16SrRNA gene were found to be *Bacillus paramycoides*, *Bacillus amyloliquefaciens and Bacillus pumilus respectively*. The plant growth-promoting rhizobacteria (PGPR) isolated from saline soil can overcome the detrimental effects of salt stress on plants. Also, PGPR bacteria can positively impact physiological functions of plants such as growth and yield and overcome disease resistance. Therefore, application of microbial inoculants to alleviate stresses and enhance yield in plants could be a low-cost and environmentally friendly option for the management of saline soil for better crop productivity.

## Supplementary Information

**Additional file 1: Fig. S1 a.** Formation of clear zone around the isolate HB6P2 represents the positive phosphate solubilization activity while isolates **b.** HB4A1 **c.** HB3A1 **d.** HB5N2 **e.** HB8P1 found negative for phosphate solubilization activity. **Fig. S2** Change in the color of filter paper on the lid of the plate from deep yellow to orange -brown represents the positive HCN production. Positive HCN production by isolates **a.** HB6J2 **b.** HB6P2 **c.** negative HCN control (un-inoculated). **Fig. S3** BLAST- N of PCR amplified 16Sr RNA gene sequence of HB6J2 with published sequences of NCBI database. **Fig. S4** BLAST-N of PCR amplified 16Sr RNA gene sequence of HB8P1 with published sequences of NCBI database. **Fig. S5** BLAST-N of PCR amplified 16Sr RNA gene sequence of HB4N3 with published sequences of NCBI database.

## Data Availability

Additional data are provided as supplementary material.

## Abbreviations

PGPR: Plant growth-promoting rhizobacteria
HCN: Hydrogen cyanide
IAA: Indole acetic acid
ICAR-CSSRI: Indian Council of Agricultural Research–Central Soil Salinity Research Institute
