# Supplementary material for: Isolation and characterization of salt-tolerant bacteria with plant growth-promoting activities from saline agricultural fields of Haryana, India
Source: J Genet Eng Biotechnol. 2021 Jun 28;19:99. doi: 10.1186/s43141-021-00186-3 (PMC8239113; doi:10.1186/s43141-021-00186-3)
Supplement: Supplementary file 1 — Additional file 1: Fig. S1 a. Formation of clear zone around the isolate HB6P2 represents the positive phosphate solubilization activity while isolates b. HB4A1 c. HB3A1 d. HB5N2 e. HB8P1 found negative for phosphate solubilization activity. Fig. S2 Change in the color of filter paper on the lid of the plate from deep yellow to orange -brown represents the positive HCN production. Positive HCN production by isolates a. HB6J2 b. HB6P2 c. negative HCN control (un-inoculated). Fig. S3 BLAST- N of PCR amplified 16Sr RNA gene sequence of HB6J2 with published sequences of NCBI database. Fig. S4 BLAST-N of PCR amplified 16Sr RNA gene sequence of HB8P1 with published sequences of NCBI database. Fig. S5 BLAST-N of PCR amplified 16Sr RNA gene sequence of HB4N3 with published sequences of NCBI database. [file 43141_2021_186_MOESM1_ESM.docx]

**Annexure –I Supplementary material**


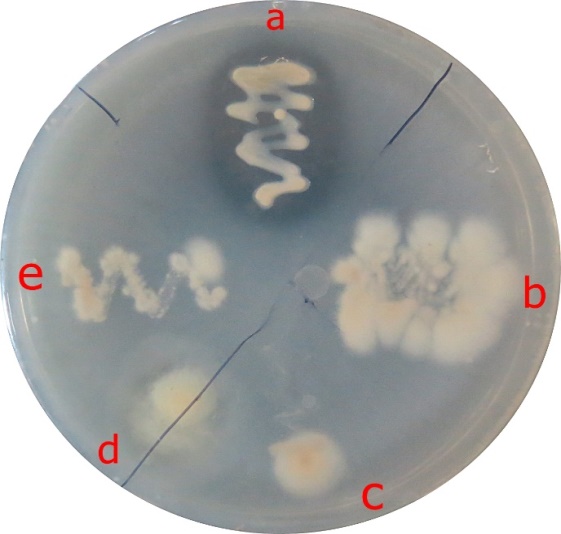


**Fig. S1 a.** Formation of clear zone around the isolate HB6P2 represents the positive phosphate solubilization activity while isolates **b.** HB4A1 **c.** HB3A1 **d.** HB5N2 **e.** HB8P1 found negative for phosphate solubilization activity.


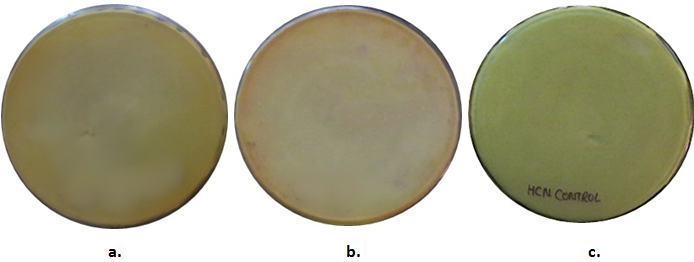


**Fig. S2** Change in the color of filter paper on the lid of the plate from deep yellow to orange -brown represents the positive HCN production. Positive HCN production by isolates **a.** HB6J2 **b.** HB6P2 **c.** negative HCN control (un-inoculated).


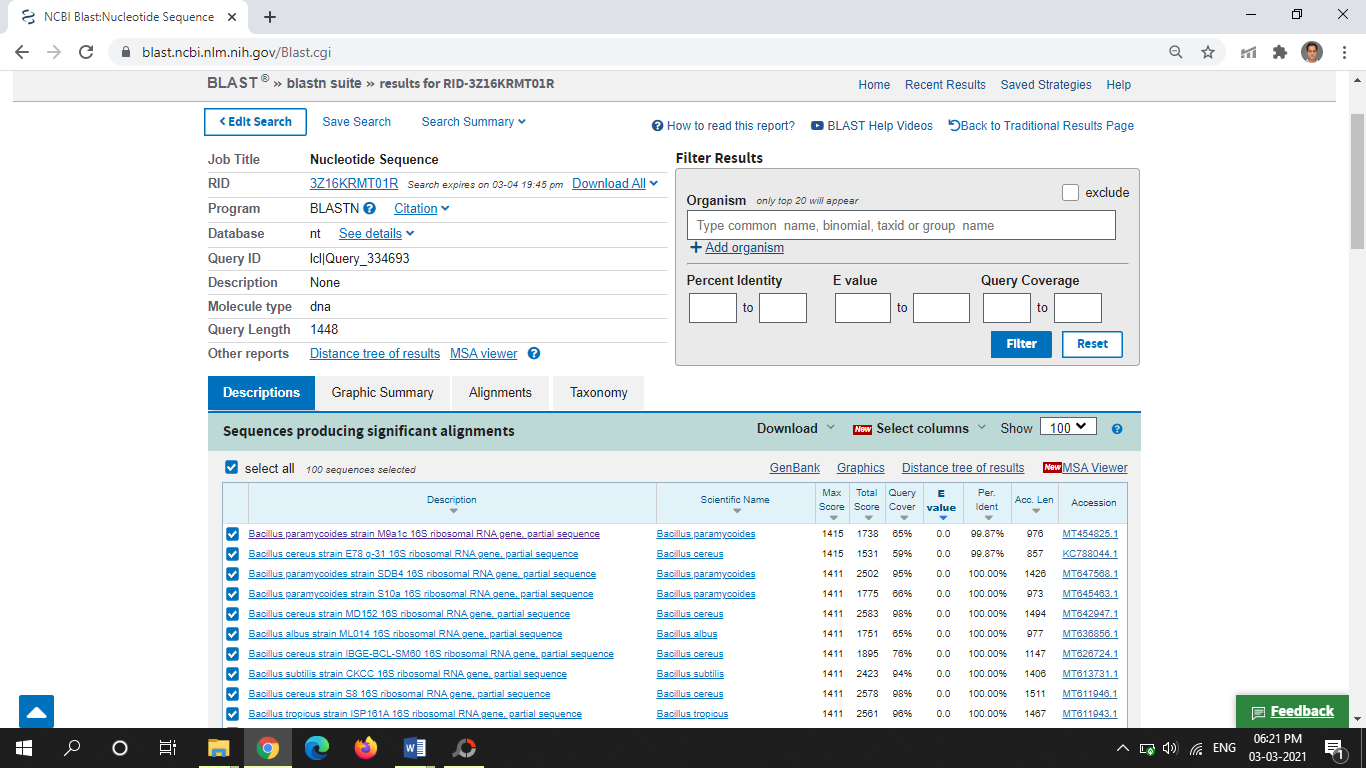


# Fig. S3 BLAST- N of PCR amplified 16Sr RNA gene sequence of HB6J2 with published sequences of NCBI database.


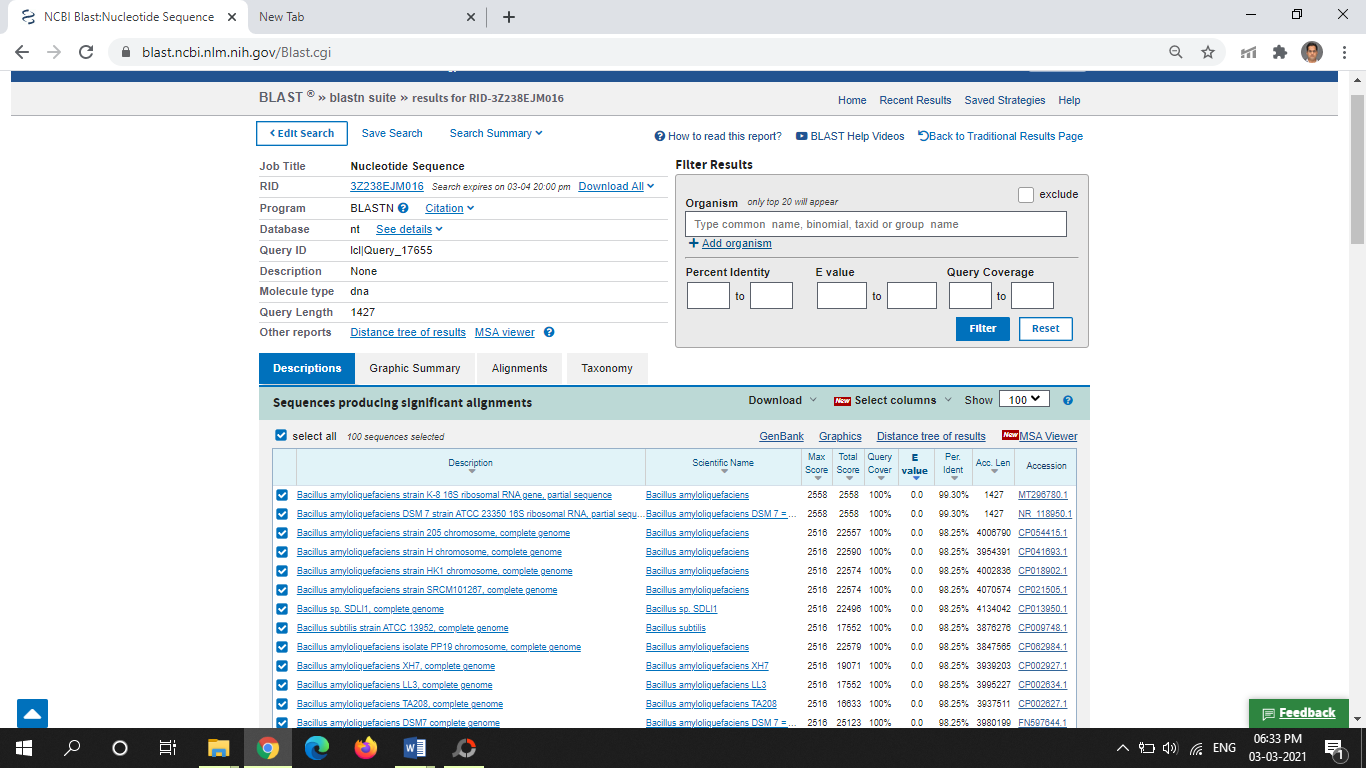


# Fig. S4 BLAST-N of PCR amplified 16Sr RNA gene sequence of HB8P1 with published sequences of NCBI database.


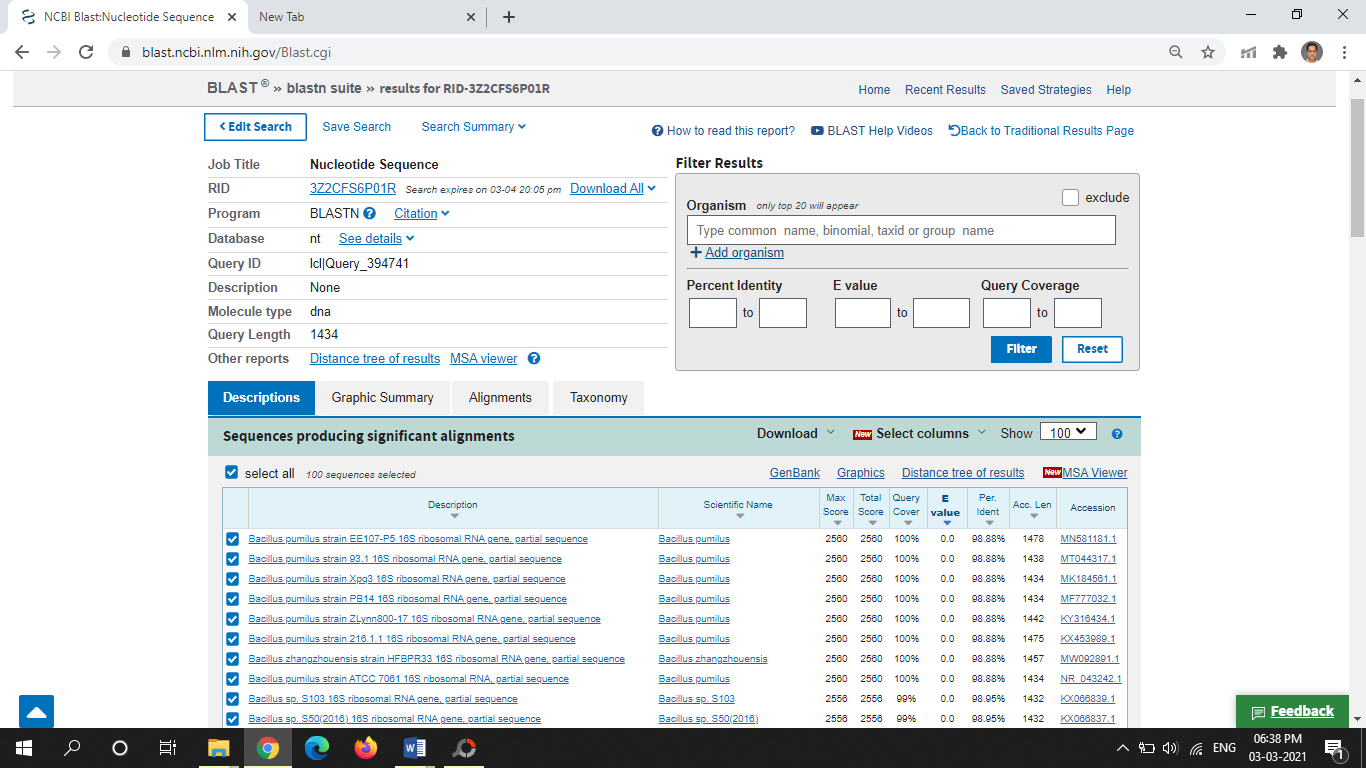


# Fig. S5 BLAST-N of PCR amplified 16Sr RNA gene sequence of HB4N3 with published sequences of NCBI database.
